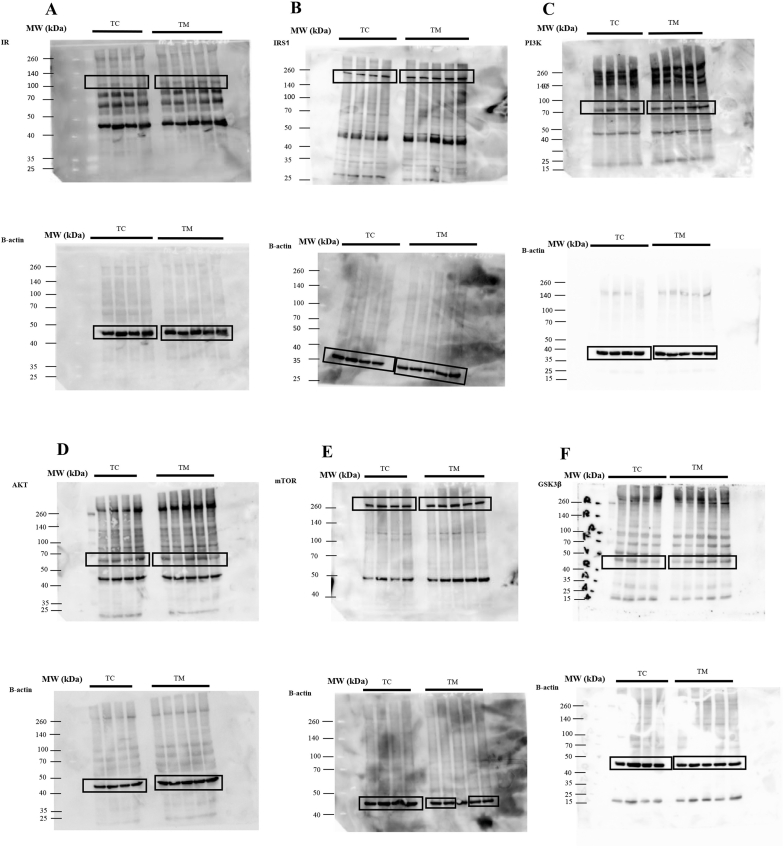# Effects of chronic treatment with metformin on brain glucose hypometabolism and central insulin actions in transgenic mice with tauopathy

**DOI:** 10.1016/j.heliyon.2024.e35752

**Published:** 2024-08-05

**Authors:** Verónica Hurtado-Carneiro, Yannick LeBaut-Ayuso, Esther Velázquez, Cinthya Flores-Lamas, Rubén Fernández-de la Rosa, Luis García-García, Francisca Gómez-Oliver, Juan Miguel Ruiz-Albusac, Miguel Ángel Pozo

**Affiliations:** a Department of Physiology, Faculty of Medicine, Complutense University, Madrid, Spain; b Department of Biochemistry and Molecular Biology, Faculty of Medicine, Complutense University, Madrid, Spain; c Pluridisciplinary Institute, Complutense University, IdISSC, Madrid, Spain; d Department of Pharmacology, Pharmacognosy and Botany, Faculty of Pharmacy, Complutense University, Madrid, Spain

**Keywords:** Brain, Glucose hypometabolism, Insulin resistance, Tauopathy, Transgenic mice

## Abstract

Brain glucose hypometabolism and insulin alterations are common features of many neurological diseases. Herein we sought to corroborate the brain glucose hypometabolism that develops with ageing in 12-months old Tau-VLW transgenic mice, a model of tauopathy, as well as to determine whether this model showed signs of altered peripheral glucose metabolism. Our results demonstrated that 12-old months Tau mice exhibited brain glucose hypometabolism as well as basal hyperglycemia, impaired glucose tolerance, hyperinsulinemia, and signs of insulin resistance. Then, we further studied the effect of chronic metformin treatment (9 months) in Tau-VLW mice from 9 to 18 months of age. Longitudinal PET neuroimaging studies revealed that chronic metformin altered the temporal profile in the progression of brain glucose hypometabolism associated with ageing. Besides, metformin altered the content and/or phosphorylation of key components of the insulin signal transduction pathway in the frontal cortex leading to significant changes in the content of the active forms. Thus, metformin increased the expression of pAKT-Y474 while reducing pmTOR-S2448 and pGSK3β. These changes might be related, at least partially, to a slow progression of ageing, neurological damage, and cognitive decline. Metformin also improved the peripheral glucose tolerance and the ability of the Tau-VLW mice to maintain their body weight through ageing. Altogether our study shows that the tau-VLW mice could be a useful model to study the potential interrelationship between tauopathy and central and peripheral glucose metabolism alterations. More importantly our results suggest that chronic metformin treatment may have direct beneficial central effects by post-transcriptional modulation of key components of the insulin signal transduction pathway.

## 1 Introduction

The term “tauopathy” includes a group of neurodegenerative disorders characterized by aberrant tau deposits in the brain. Currently, there are more than 26 types that have been classified as primary, when tau is the main responsible agent in the pathology and, as secondary, when tau aggregation is a consequence of other pathological events, such as amyloid beta (Aβ) deposition in Alzheimer's disease (AD) [1]*.* Besides, a relationship between neurodegenerative and metabolic disorders such as Type 2 Diabetes Mellitus (T2DM) is supported by epidemiological, preclinical and clinical evidence. These disorders share some pathological commonalities, such as insulin signalling abnormalities [[2], [3], [4], [5]] which has taken a predominant stance as a risk factor for developing dementia, both in AD and T2DM patients [[6], [7], [8]]. This association has presented itself as an opportunity to repurpose antidiabetic drugs for the potential promising treatment of neurodegenerative disorders. Furthermore, the manifestation of memory loss as well as cognitive and executive decline happens between 15 and 20 years after the onset of these pathologies offering a wide time window for intervention [9,10].

Currently, the most promising therapies for T2DM include 3 pharmacological groups: (i) insulin-sensitizing agents such as metformin and peroxisome proliferator-activator receptor gamma (PPARγ) agonists; (ii) insulin mimetic molecules such as glucagon-like peptide-1 (GLP-1) and (iii) insulin secretagogues analogues. Among them, metformin is the most widely used as an initial oral glucose-lowering monotherapy medication for DMT2 [11]. Metformin is a biguanide that improves fasting insulin levels, having insulin sensitizing properties and hepatic glucose production induced by AMP-activated protein kinase (AMPK) activation [12,13]. Furthermore, metformin crosses the blood brain barrier (BBB) [14] and studies in humans have reported that metformin improves cognitive dysfunction in AD patients [15] also reducing the incidence of dementia in diabetic patients [16]. Likewise, preclinical studies also report that metformin prevents memory impairment in streptozotocin-induced diabetic mice [17]. At the molecular level, metformin has been shown to prevent the increase of phosphorylated-tau (p-tau) protein expression in the parietal cortex and the accumulation of Aβ plaque in the hippocampus [17]. It also reduces the expression of both total tau and p-tau in the hippocampus of a murine model of leptin-resistant obese mice [18]. Similarly, studies in primary neurons from transgenic mice overexpressing human tau have shown that metformin reduced tau phosphorylation by induction of phosphatase activity [19]. In amyloid precursor protein/presenilin 1 (APP/PS1) mice, 8 weeks of metformin treatment reduced amyloid plaque deposition and brain hypometabolism [20]. Furthermore, in this same APP/PS1 mouse model, metformin treatment rescued spatial memory, prevented neuronal cells death, increased adult hippocampal neurogenesis, inhibited the inflammatory response, reduced proinflammatory cytokine levels, restored the antioxidant status and increased the protein levels of both *p*-AMPK and insulin-degrading enzyme (IDE), altogether contributing to an improvement in cognitive impairment [20,21]. Besides, metformin has been shown to ameliorate neuronal insulin resistance in an *in vitro* neuronal insulin-resistant model also showing hallmark AD-like changes [22] and to inhibit neuronal damage by upregulating GLP-1 receptor (GLP1R) [23].

However, even though metformin seems to attenuate the progression of various ageing-related diseases, including AD [24], other studies have reported conflicting results regarding its benefits. Thus, the long-term use of this antidiabetic drug increases the risk of AD [25,26]. In fact, although the combination of metformin and insulin reduces Aβ aggregation, metformin alone has been shown to increase the production of both intracellular and extracellular Aβ species without affecting their degradation [27,28], promoting insoluble tau aggregation [29], and increasing the cognitive impairment in patients with diabetes [30]. Taken together, this suggests that the risk of tauopathy could be increased in diabetic patients chronically treated with metformin [30]. Finally, as is common for all drugs, metformin has side effects in some patients causing vitamin B12 deficiency, epidermal growth factor (EGF) reduction, and tumour necrosis factor (TNF) increase [31,32].

Taking into account the aforementioned effects of metformin modulating the progression of neurodegenerative diseases [33,34], we consider that it is worthwhile to extend the study of the effects of this antidiabetic drug. Thus, we sought to evaluate the longitudinal effects of chronic metformin treatment on brain glucose metabolism, as well as to study its effect after long-term treatment on key players of the insulin signalling pathway, which are known to be altered both in patients with AD and in two lines of animal models with preclinical AD [35,36]. First, we wanted to corroborate the reported brain glucose hypometabolism characteristic of the transgenic tau-VLW, a mouse model characterised by overexpression of p-tau by using positron emission tomography (PET) with [^18^F]fluorodeoxyglucose ([^18^F]FDG) [37]. We also studied whether this model showed signs of peripheral insulin resistance. After confirming that the mice model met the criteria by reflecting both central and peripheral metabolic abnormalities, we performed a longitudinal study evaluating the effects of chronic metformin treatment on brain glucose metabolism from 9 to 18 months of age. In addition, we also evaluated gene expression and protein content (both as total and as phosphorylated forms) of key insulin signalling mediators in the frontal cortices of the tau-VLW transgenic mice at the end of the metformin treatment.

## 2 Material and methods

### 2.1 Animals

Tau-VLW transgenic mice, characterised as a model of frontotemporal dementia (FTD), a primary tauopathy, were used. This transgenic mouse overexpresses the phosphorylated human Tau (P-hTau) protein [38,39] carrying three mutations G272**V**, P301**L** and R406**W**, associated with the modification of the microtubule-associated protein tau (MAPT) gene, which encodes tau protein under the control of the Thy-1 promoter [40]. The animals were generously donated by Prof. Jesús Ávila, Center of Molecular Biology “Severo Ochoa” (CSIC-UAM), Madrid, Spain. Wild-type (WT) animals used as control were obtained from the same breeding colonies and were housed in the same facilities. All mice were housed in standard cages (3–4 mice/cage), on a ventilated rack (Tecniplast, Italy) under controlled temperature (22 ± 2 °C), a 12 h light/dark cycle (8:00 a.m.-8:00 p.m.) and with free access to standard rodent chow and tap water. Food was only removed for the 12 h before the different tests, including the [^18^F]FDG-PET acquisitions to reduce competition between plasma glucose and the radiotracer for the glucose transporters.

In the first study (see Fig. 1), adult male WT (n = 10) and Tau-VLW (n = 12) mice were used, being 9 months old at the beginning of the experiments. All tests were performed at 12 months of age and then the animals were sacrificed.

**Fig. 1:**
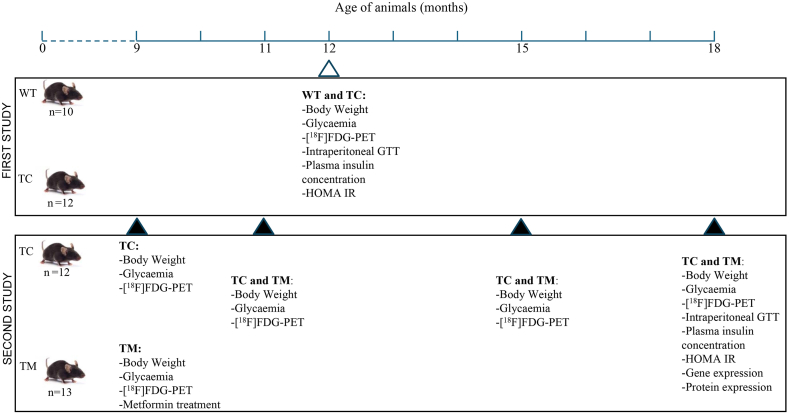
Schematic figure showing the time-course and experimental interventions corresponding to the two studies performed. First study 1: wild type (WT), Tau control (TC); Second study 2: TC and Tau metformin (TM).

As depicted in Fig. 1, in the second study only adult Tau-VLW transgenic mice were used. The general housing conditions were comparable to those in the previous experiment, with the exception that the animals were divided into two experimental groups: control (TC, n = 12) and chronically treated with metformin (TM, n = 13). The tests were carried out at 9, 11, 15 and 18 months of age and only at this age were the animals sacrificed.

The study was approved by the Animal Research Ethics Committee of the Complutense University of Madrid, and it was carried out in accordance with regulations of the European Union (2010/63/UE) and Spain (RD53/2013) regarding animal welfare.

### 2.2 Experimental studies and procedures

In the first study, we performed a [^18^F]FDG-PET neuroimaging study aimed to corroborate the previously reported brain glucose hypometabolism in the Tau-VLW mice [37]. To this end, 12 months-old male transgenic tau-VLW and WT mice were used. The age of 12 months was selected because brain glucose hypometabolism in the Tau-VLW model was reported to start at 11 months of age [37]. We also studied whether, in comparison with the WT, the transgenic Tau-VLW mouse model showed signs of glucose homeostasis alteration by evaluating the glucose response to Intraperitoneal Glucose Tolerance Test (IpGTT), plasma insulin concentrations and the homeostasis model assessment index (HOMA-IR).

After confirming that the transgenic mouse model of tauopathy showed central and peripheral glucose metabolism alterations, we performed a second study focussed on the eventual effects of chronic (9 months) metformin treatment on brain glucose metabolism along the lifespan of tau-VLW mice (from 9- to 18 months-age). To this end, tau-VLW mice were divided into two experimental groups: control (TC) and metformin-treated group (TM). For the TM group, metformin was diluted in the drinking water at a final concentration of 1 mg/mL. During the experiment, both TC and TM mice drank a daily mean volume of approximately 3 mL resulting in a mean daily dose of metformin of 100 mg/kg. All mice underwent longitudinal [^18^F]FDG-PET neuroimaging studies at 9 months of age (baseline, before initiating the metformin treatment, TC, n = 12; TM, n = 13) as well as at the ages of 11 (TC, n = 12; TM, n = 12), 15 (TC, n = 10; TM, n = 11), and 18 months (TC, n = 7; TM, n = 11) of age (after 2, 6 and 9 months of treatment, respectively). The reduction in the “n” in some groups was due to death of the animals due to the natural causes associated to the aging process. As we did in the first experiment, the glucose response to IpGTT, plasma insulin concentrations and the HOMA-IR were evaluated. At the end of the experiment (at 18 months of age) after 9 months of metformin treatment, the animals were sacrificed, the brains were collected, and the cortices were dissected and processed for the analysis of gene expression and protein content (both total and phosphorylated forms) of key mediators of the insulin signalling pathway.

#### 2.2.1 [^18^F]FDG-PET neuroimaging and brain glucose metabolism quantification

To evaluate brain metabolic activity, [^18^F]FDG-PET imaging was carried out by using a small-animal dedicated PET/CT (computed tomography) scanner (Albira, Bruker Corporation, USA) as previously reported [41]. Before image acquisition, mice were fasted overnight. Immediately prior to intraperitoneally (i.p.) injection of FDG (approximately 5600 kBq, Curium Pharma Madrid, Spain, in 0.2 mL of 0.9 % NaCl), plasma glucose was measured in blood obtained from the tail. Forty-five min after radiotracer injection, mice were anesthetized with isoflurane and underwent 20-min PET followed by CT acquisition. The tomographic images were then reconstructed using the maximum likelihood expectation maximization (MLEM) and filtered back projection (FBP) algorithms for the PET and CT images, respectively. The final reconstructed PET image resulted into 160 × 160 x 189 pixels (0.5 mm of pixel size). All PET data underwent corrections for dead time, normalization, decay and attenuation corrections. For quantification of the regional brain metabolism, both PET and CT images were co-registered to a magnetic resonance image (MRI) mouse brain template, containing the main brain areas. To account for the body weight variations and the administered FDG doses, the standardised uptake value (SUV) was calculated. The SUV is a quantitative measure to evaluate the concentration of FDG within a specific region of interest and it is the most widely used parameter for evaluating brain glucose metabolism in both human and experimental animals by FDG-PET studies [42,43]. All the tasks described above were carried out using PMOD software (PMOD Technologies Ltd., Zurich, Switzerland).

#### 2.2.2 Blood glucose response to Intraperitoneal Glucose Tolerance Test (IpGTT)

12-month-old WT (n = 8) and Tau-VLM (n = 10) mice (in the first experiment) and 18-month-old control (n = 6) and metformin (n = 9) Tau-VLM mice (in the second experiment) were fasted overnight and then i.p. injected with glucose (2 g/kg of BW, with a final concentration of 60 mg/100 μl) using saline as vehicle [44]. Blood samples were taken by tail venesection at 0 min (basal, just before glucose injection) and at 15, 30, 60, 90, 120 and 150 min after the i.p. glucose load. Mice had free access to water during the testing period. Blood glucose concentrations were measured with a glucometer (GlucoMen aero 2K, A. Meranini Diagnostics).

#### 2.2.3 Animal sacrifice and samples collection

Mice were sacrificed by cervical dislocation at 12 months of age in the first experiment and at 18 months in the second one. Immediately after cervical dislocation, mice were decapitated, and trunk blood was collected. Plasma was obtained by mixing 300 μL of blood with 30 μL of 1.2 mg/mL Aprotinin-EDTA and centrifuged at 4000 rpm for 10 min at 4 °C. Supernatants were kept at −80 °C until further analyses. Brains were removed and frontal cortices dissected and stored at −80 °C until the analyses.

#### 2.2.4 Quantification of plasma insulin concentrations

Insulin resistance is usually accompanied by increased basal plasma insulin concentrations. Consequently, plasma insulin concentrations were determined in 12-month-old (WT, n = 9; TC, n = 3) and 18-month-old mice (TC, n = 3; TM, n = 4) that were fasted overnight and then sacrificed. Plasma insulin concentrations were measured by means of a competitive ELISA Kit (Mouse Insulin Elisa kit, Thermo Fisher), following the manufacturer's instructions. The absorbance was measured by using the Synergy HTX multi-mode reader (BioTek) at a wavelength of 450 nm. The values were obtained based on a standard curve of known hormone concentrations.

#### 2.2.5 Determination of the homeostasis model assessment index (HOMA-IR) as a surrogate for insulin resistance

To assess insulin sensitivity we use the HOMA-IR mathematical model according to the following equation:

HOMA IR = [Fasting blood glycemia (mg/dL) × Fasting blood insulinemia (μUI/mL)]/405.

Ranges of insulin for this index are: <1.0, good insulin sensitivity, >1.9, early insulin resistance, >2.9, significant insulin resistance [45].

#### 2.2.6 RNA samples extraction from cortices and real-time quantitative polymerase chain reaction (RT-PCR)

In the second study, total RNA from the dissected cortices (50–100 mg) corresponding to 18-month-old TC (n = 5) and TM (n = 6) mice was extracted with TRIzol reagent (Life Technologies, Barcelona, Spain). RNA integrity was tested with the Bioanalyzer 2100 (Agilent) and cDNA synthesis was performed using the high-capacity cDNA archive kit (Applied Biosystems), using 2 μg of RNA as template, following the manufacturer's instructions. Four microlitres of a 1:10 dilution of the cDNA was used as a template for the PCR. TaqMan® Fast Advance Assay (Applied Biosystems, Foster City, CA) was used to quantify mRNA levels by RT-PCR on a StepOnePlusTM Real-Time PCR System (Applied Biosystems). Details of the primers and probes used are listed in Table 1. The PCR conditions were 95 °C for 20 s, followed by 40 cycles at 95 °C for 1 s, and then at 60 °C for 20 s. The data were analysed using 7300 System SDS software (version 1.2.3, 2004). The 2^−ΔΔCt^ method was used to quantify the relative mRNA levels. β-actin housekeeping gene was used for normalization.

**Table 1:** Identification of primers used in the different gene expression assays.

Gene	Probe identification (Taqman® Assay)
Insr (IR)	Mm01211875_m1
Irs1 (IRS-1)	Mm01278327_m1
Akt (AKT)	Mm 01331626_m1
Pik3r1 (PI3K)	Mm 01282781_m1
Gsk3β (GSK3β)	Mm 00444911_m1
Mtor (mTOR)	Mm 00444968_m1
Actb (ACTB)	Mm 00607939_s1

IR, insulin receptor; IRS-1, insulin receptor substrate 1; AKT (also known as AKT/PKB, protein kinase B; PI3K, phosphatidylinositol 3-kinase; GSK3β, Glycogen synthase kinase-3 beta; mTOR, mammalian Target of Rapamycin; ACTB, Beta-actin.

#### 2.2.7 Brain frontal cortical protein content analysis by western blot

For the analysis of protein content by Western blot, frontal cortical pieces 18-month-old TC (n = 4–5) and TM (n = 4–5) mice were lysed in RIPA buffer (PBS, 1 % NP-40, 0.5 % sodium deoxycholate, 1 mM PMSF, 10 mg/mL leupeptin, 1 mM Na_2_VO_4_, 2.5 mM Na_4_P_2_O_7_ and 10 mM NaF) in the presence of a cocktail of protease inhibitors (Roche Diagnostics, Mannheim, Germany) and immediately exposed to wave irradiation for 5 s [46]. Tissue homogenization was performed with syringe needles of different calibres. Thereafter, the samples were centrifuged at 14000 rpm for 5 min to remove insoluble debris. Protein concentrations were determined in the supernatants by the Lowry method (DCTM Protein Assay, BioRad) and measured by the Synergy HTX multi-mode reader (BioTek). Lysates were diluted in Laemmli buffer (Tris-HCL 277.8 mM, pH 6.8, 4.4 % SDS, 44.4 % glycerol, 5 % β-mercaptoethanol, 0.02 % bromophenol blue) to a final concentration of 1–2 μg/μL and then boiled for 5 min at 100 °C. Before starting the electrophoretic procedure, 15–20 μg of protein were loaded onto 10 % polyacrylamide gels (TGX Stain-Free Cast Acrylamide Kit, BioRad). Electrophoresis was carried out with buffer (Tris 25 mM, 192 mM Glycine and 0.01 % SDS) at 120 V. Afterwards, the protein samples were transferred to a PVDF membrane (Immun-Blot® PVDF, Bio-Rad) and the membrane was blocked for 1 h in a TBS-Tween solution pH 7.6 (140 mM NaCl, 2 mM Tris, Tween 20 at 0,1 %) containing 5 % BSA, followed by overnight incubation at 4 °C with the primary antibodies (Table 2). Then, membranes were washed 3 times for 10 min with TBS-Tween and incubated with the corresponding secondary antibody for 1 h at room temperature. Chemiluminescence detection was performed using an ECL Western blotting system (Millipore Co, Darmstadt, Germany). A chemiluminescent substrate, such as ECL (Enhanced chemiluminiscence), reacts with a secondary antibody conjugated to the HRP enzyme, producing a signal, which is captured on X-ray films (X Ray Film Medical Plus, Sakura Konica Minolta) according to the manufacturer's instructions and developed in an Agfa Curix 60 developing equipment. The exposure time will depend in each case on the detected signal. Subsequently, the autoradiographs were analysed by densitometry, ChemiDoc Imagine System REF: #12003153 Bio-Rad) and Quantified (Image Lab 6.0 software, Bio-Rad), according to manufacturer's instructions.

**Table 2:** Antibodies used for protein detection by Western Blot.

Antibody	Host	Manufactured
Anti β-IR	Mouse	EMD Millipore - Sigma-Aldrich
Anti p-IR-Y972	Rabbit	Millipore
Anti IRS1	Rabbit	BioRad
Anti p-IRS1-Ym608/h612	Rabbit	Sigma-Aldrich
Anti AKT	Rabbit	Sigma-Aldrich
Anti *p*-AKT-Y474	Rabbit	Sigma-Aldrich
Anti mTOR	Rabbit	BioRad
Anti *p*-mTOR-S2448	Rabbit	Sigma-Aldrich
Anti PI3Kα	Rabbit	EMD Millipore - Sigma-Aldrich
Anti p-PI3K p85-(Y458)/p55-(Y199)	Rabbit	EMD Millipore - Sigma-Aldrich
Anti GSK3β	Mouse	BioRad
Anti *p*-GSK3β-Y216	Rabbit	Sigma-Aldrich
Anti β-actin	Mouse	Sigma-Aldrich
Anti Mouse	Goat	Sigma-Aldrich
Anti Rabbit	Goat	Sigma-Aldrich

*β-IR*, *insulin receptor beta-subunit; p-, phospho; p85/p55, alpha/gamma regulatory subunits of PI3K. All antibodies were used diluted 1:1000*.

### 2.3 Statistical analysis

Data were analysed with SPSS v.24 software. Student t-tests were used to compare brain glucose metabolism data obtained by PET neuroimaging between 12-months old WT and Tau-VLW mice in the first study, as well as in the second study when assessing the statistical effects of chronic metformin treatment on brain glucose metabolism in TC and TM within time. The effects of time (9- to 18-months of age) within TC and TM mice were evaluated using the nonparametric Wilcoxon signed rank test for repeated measures.

Blood glucose concentration data were analysed using one-way analysis of variance (ANOVA) or two-way ANOVA as appropriate. When the ANOVA was significant, individual differences were evaluated using post-hoc tests. Thus, Dunnet's post hoc test were used to compare, within group, the effect of time with their respective control (time 0). Bonferroni post hoc tests were used to compare glucose concentrations between groups within time.

Data for plasma insulin concentrations, HOMA-IR index, and RNA and protein content in the cerebral cortex were analysed using Student's unpaired *t*-test. In all cases, statistical significance was considered when p < 0.05. A trend to significance was considered when 0.05< p < 0.1.

The initial sample sizes for the different experiments were calculated with a minimum confidence level of 95 % and an estimation error of the relative fraction of the sample of 20 % in relation to the relative fraction of the population, whose order of magnitude was estimated between 10 and 40 %. The confidence intervals of the means of each group, as well as those of the differences between the means of the different groups, were calculated for various alpha levels of significance. Effect sizes were calculated using Cohen's d. Taking the above into account, the minimum sample sizes were calculated for confidence levels between 95 % (α = 0.05) and 99.9 % (α = 0.001) and negative errors between 20 % (β = 0.2; statistical power = 80 %) and 1 % (β = 0.01; statistical power = 99 %). In all the results that proved to be statistically significant, the number of mice used was adequate.

Data are shown as mean ± SEM, the number of animals in each experimental group being indicated in the corresponding experimental procedures section and in the figure legends.

## 3 Results

### 3.1 Characterization of 12-months-old transgenic Tau-VLW *vs* age-matched WT

**Fig. 2:**
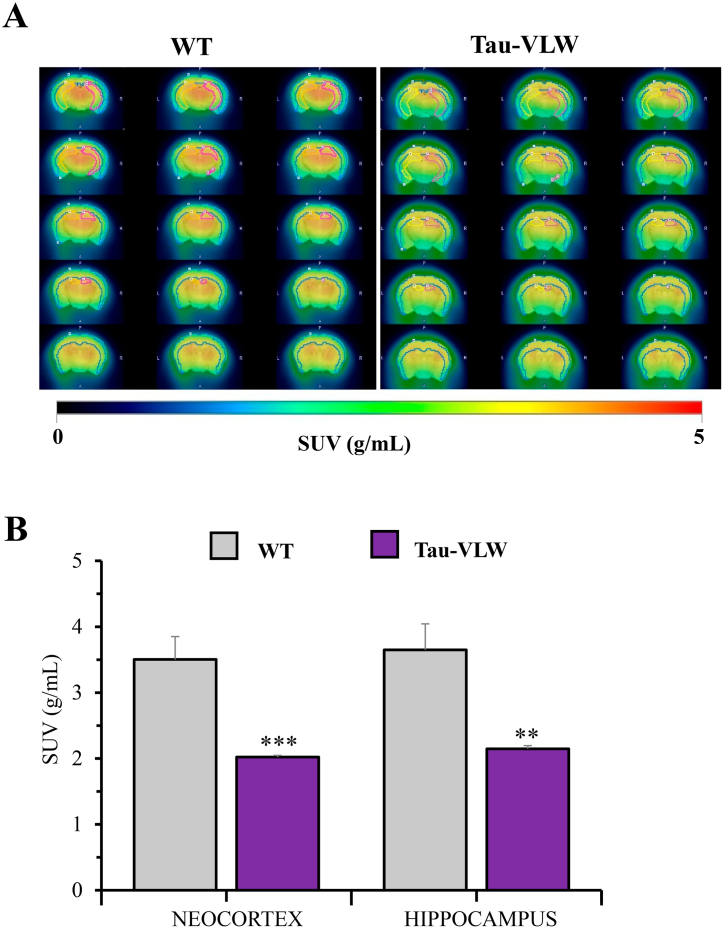
12-months-old transgenic Tau-VLW mice are characterized by brain glucose hypometabolism. **(A)** Representative images depicting brain [^18^F]FDG uptake in Tau-VLW (white bars) vs age-matched WT mice (striped bars). **(B)** [^18^F]FDG-PET neuroimaging quantification is shown as SUV in the cortex and in the hippocampus. Data are presented as mean ± SEM (n = 7/group). Unpaired Student's t-test was used to analyse significant differences. **p < 0.01, ***p < 0.001.

Herein one of our two main objectives was to confirm the previously reported brain glucose hypometabolism characteristic of the Tau-VLW mice that started at 11 months of age becoming more prominent as the animals aged [37]. As shown in Fig. 2A, [^18^F]FDG uptake was lower in the examined brain areas of 12-month-old Tau-VLW than in WT mice. The brain glucose hypometabolism reached high significance and high effect sizes in the cortex (p = 0.0008, d = 2.572) and in the hippocampus (p = 0.0016, d = 2.269) (Fig. 2B).

**Fig. 3:**
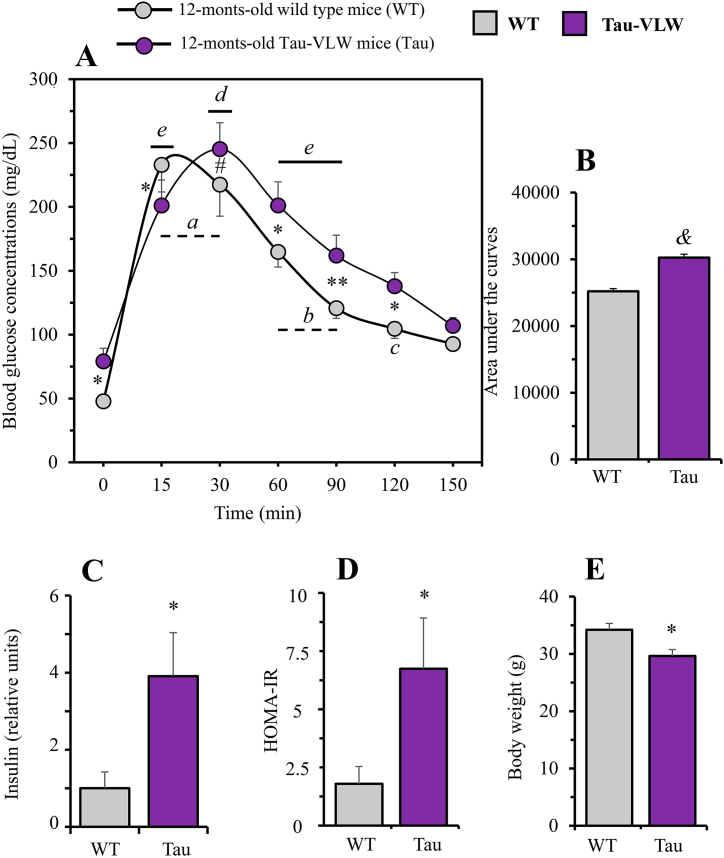
Evaluation of peripheral markers of glucose metabolism in 12-months-old wild type and Tau-VLW mice. (A) Intraperitoneal glucose tolerance test (IpGTT) on overnight fasted mice and (B) Area under the curves. Blood glucose concentrations under basal conditions (time 0, before 2 g/kg BW glucose overload) as well as 15, 30, 60, 90, 120 and 150 min later are shown. Values are expressed as means ± SEM (WT, n = 8; Tau, n = 10). Significant differences between WT and Tau within time are depicted as *p < 0.05, **p < 0.01. The trend to significance #p = 0.08 is also shown (two-way ANOVA followed by post-hoc Bonferroni correction). Significant differences when compared with time 0 within WT (^a^p<0.001, ^b^p < 0.01, the trend to significance ^c^p = 0.06 is also shown) and, within Tau (^d^p < 0.001, ^e^p < 0.01) (one-way ANOVA followed by Dunnett's correction for comparisons with time 0). ^&^*p* < 0.0001, when compared area under the curves **(C) Fasting plasma insulin concentrations and (D) HOMA-IR index.** (WT, n = 9; Tau, n = 3). **(E) Body weight.** (WT, n = 8; Tau, n = 10). All values are expressed as means ± SEM. Unpaired Student's t-test was used to analyse significant differences. *p < 0.05, when comparing WT with Tau mice.

Once our previous data were confirmed [37], we focussed on the second objective, the assessment of glucose homeostasis in 12-months-old WT and transgenic Tau-VLW by studying glucose tolerance using the IpGTT, fasting concentrations of insulin and evaluating insulin resistance from the surrogate measure of HOMA-IR (Fig. 3).

Fig. 3A shows that the IpGTT produced a highly significant time-dependent increase in blood glucose concentrations in both WT (F = 23.85, *p* < 0.0001) and Tau-VLW (F = 14.16, *p* < 0.0001) mice. Glucose concentrations in WT mice peaked at 15 min (*p* < 0.001, compared to time 0) whereas in Tau mice the peak was delayed up to 30 min (*p* < 0.001). Glucose concentrations decreased in both groups at 90 min but without reaching basal concentrations (*p* < 0.01, compared to time 0). Basal glucose concentrations were finally reached at 120 and 150 min. Likewise, the difference in the effect of glucose overload between WT and Tau mice was highly significant (F = 29.50, *p* < 0.0001), being greater in Tau mice than in WT mice measured either by the effect size (d = 3.5) or by comparing their area under the curves (*p* < 0.0001; Fig. 3B). As shown, basal blood glucose was significantly lower in WT than in Tau-VLW (*p* < 0.05) and, except at 15 min (*p* < 0.05), circulating glucose concentrations tended to remain significantly higher in Tau than in WT mice, reaching statistically significance at 60 (*p* < 0.05), 90 (*p* < 0.01) and 120 min (*p* < 0.05). Besides, both the basal blood insulin concentrations (Fig. 3C) and the HOMA-IR index (Fig. 3D) were significantly higher in Tau mice compared to WT mice (p = 0.0123, d = 2.11 and p = 0.0178, d = 1.89; respectively). In addition, at 12 months of age, WT mice were significantly heavier (p = 0.0151, d = 1.602) than their Tau counterparts (Fig. 3E).

### 3.2 Characterization of the effects of chronic metformin treatment in Tau-VLW mice

**Fig. 4:**
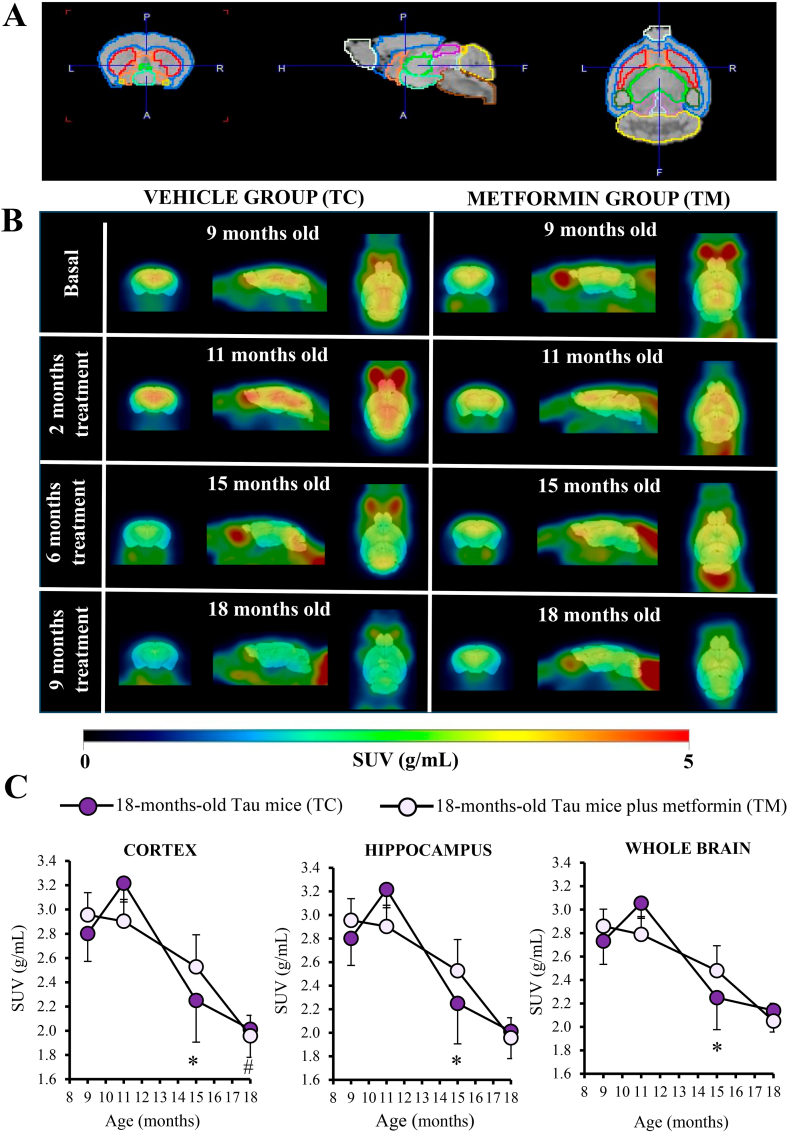
Effect of chronic metformin treatment on brain glucose metabolism in Tau-VLW transgenic mice. (A) Coronal, transaxial and sagittal views of the mouse MRI brain template with the regions of interests (ROIs) corresponding to the main brain areas. (B) Representative [^18^F]FDG-PET images in SUV scale (coronal, transaxial and sagittal planes) of TC (left panel) and TM (right panel) from 9- to 18 months-old. (C) [^18^F]FDG-PET SUV changes in cortex, hippocampus and whole brain. Data are shown as mean ± SEM (TC, n = 12, 12, 10 and 7 at 9, 11, 15 and 18-months-old mice, respectively; TM, n = 13, 12, 11 and 11 at 9, 11, 15 and 18-months-old mice, respectively). *p < 0.05 compared to TC; ^#^*p* < 0.05 comparing TM at 18 months vs 15 months, Wilcoxon signed-rank test.

As shown in Fig. 4A–C and in Table 3, the longitudinal PET neuroimaging studies in both vehicle (TC) and chronically metformin-treated Tau-VLW (TM) mice showed an overall progressive brain hypometabolism over time, but the time dynamic was different. Thus, within the age window ranging from 11 to 15 months, the decline was more marked in the TC than in the TM mice (see Fig. 4C). TC mice significantly reduced metabolic activity (*p < 0.05) from 11 to 15 months of age in the whole brain, as well as in all the other studied regions. This effect was no longer observed within the age window ranging from 15- to 18-months. Nevertheless, from 15 to 18-months of age, TM mice significantly reduced their metabolic activity in the cortex (p = 0.033).

**Table 3:** Statistical analysis of SUV values of glucose metabolism measured by [^18^F]FDG-PET in selected brain areas.

Age range (months-old)	VEHICLE (TC)			METFORMIN (TM)		
	9 to 11	11 to 15	15 to 18	9 to 11	11 to 15	15 to 18
**Cortex**	Z = −1.10 (p = 0.272)	Z = −2.70 (p = 0.007*)	Z = −1.52 (p = 0.128)	Z = −1.06 (p = 0.289)	Z = −0.98 (p = 0.328)	Z = −2.14 (p = 0.033*)
**Striatum**	Z = −1.57 (p = 0.117)	Z = −2.55 (p = 0.011*)	Z = −1.52 (p = 0.128)	Z = −1.10 (p = 0.272)	Z = −0.71 (p = 0.477)	Z = −1.87 (p = 0.062)
**Hippocampus**	Z = −1.57 (p = 0.117)	Z = −2.80 (p = 0.005*)	Z = −1.35 (p = 0.176)	Z = −1.02 (p = 0.308)	Z = −0.89 (p = 0.374)	Z = −1.78 (p = 0.075)
**Cerebellum**	Z = −0.78 (p = 0.433)	Z = −2.81 (p = 0.005*)	Z = −0.17 (p = 0.866)	Z = −0.86 (p = 0.388)	Z = −1.33 (p = 0.182)	Z = −0.97 (p = 0.333)
**Whole brain**	Z = −1.57 (p = 0.117)	Z = −2.70 (p = 0.007*)	Z = −1.52 (p = 0.128)	Z = −0.94 (p = 0.346)	Z = −0.98 (p = 0.328)	Z = −1.60 (p = 0.109)
**n**	12	10	7	12	11	11

Z score and p-values from the Wilcoxon signed-rank test performed for each pair of measurements within each brain region. Significant differences (*) are indicated.

### 3.3 Effect of chronic metformin treatment on glucose tolerance test, blood insulin concentrations, BW gain and survival in Tau-VLW transgenic mice

**Fig. 5:**
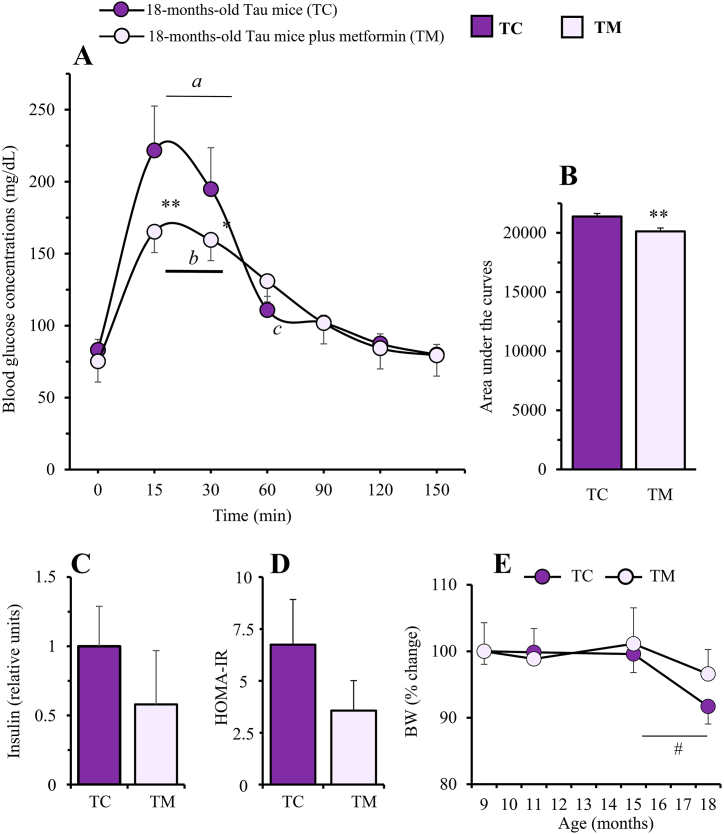
Evaluation of peripheral markers of glucose metabolism in 18-months-old control Tau-VLW mice (TC) and chronically (9 months) treated with metformin (TM). (A) Intraperitoneal glucose tolerance test on overnight fasted mice (IpGTT) and (B) Area under the curves. Blood glucose concentrations under basal conditions (time 0, before 2 g/kg BW glucose overload) as well as 15, 30, 60, 90, 120 and 150 min later. Values are expressed as means ± SEM (TC, n = 6; TM, n = 9). Significant differences when compared with time 0 within TC (^a^p<0.01) and, within TM (^b^p < 0.01) the trend to significance ^c^p = 0.06 is also shown (one-way ANOVA followed by Dunnett's correction for comparisons with time 0). Significant differences between TC and TM mice in each time point are depicted as *p < 0.05, **p < 0.01 (two-way ANOVA followed by post-hoc Bonferroni correction). **p < 0.01, when compared area under the curves (Student's unpaired *t*-test). **(C) Fasting plasma insulin concentrations and (D) HOMA-IR index.** (TC, n = 3; TM, n = 4). **(E) Cumulative body weight changes (% *vs* initial BW).** (TC, n = 12, 12, 10 and 6 at 9, 11, 15 and 18-months-old mice, respectively; TM, n = 13, 12, 11 and 11, at 9, 11, 15 and 18-months-old mice, respectively). All values are expressed as means ± SEM. ^#^*p* = 0.06 (Student's unpaired *t*-test).

As depicted in Fig. 5A, blood glucose concentration in response to the intraperitoneal administration of a glucose overload was significantly dependent on time in 18-month-old Tau-VLW mice both, control (F = 11.45, *p* < 0.0001) and chronically treated with metformin (F = 8.47, *p* < 0.0001). The glucose concentrations in both groups were significantly higher at 15–30 min (*p* < 0.01) compared with basal concentrations at time 0. This effect was not significant at other times. Likewise, the effect interaction of metformin in Tau mice after glucose overload was also highly significant (F = 17.17, *p* < 0.0001), being greater in untreated Tau mice than in those treated with metformin, measured either by the effect size (d = 1.596) or by comparing their area under the curves (p = 0.0097; Fig. 5B). Thus, although basal blood glucose concentrations were similar in both groups, they were significantly lower in metformin-treated compared to untreated animals at 15 min (*p* < 0.01) and at 30 min (*p* < 0.05). However, both basal blood plasma insulin concentrations and the HOMA-IR index showed very small (effect sizes, d = 0.62 and d = 0.97, respectively) but non-significant decreases after metformin treatment (Fig. 5C and D).

Interestingly, metformin treatment allowed mice to defend their BW throughout the whole experimental period (Fig. 5E). This effect was evident between 15- and 18 months of age when TC mice lost a 9.1 % (p = 0.06) of their initial BW while TM mice maintained their weight.

### 3.4 Effects of chronic metformin treatment on basal mRNA levels of key genes involved in the insulin signalling pathway in the frontal cortex of Tau-VLW transgenic mice

**Fig. 6:**
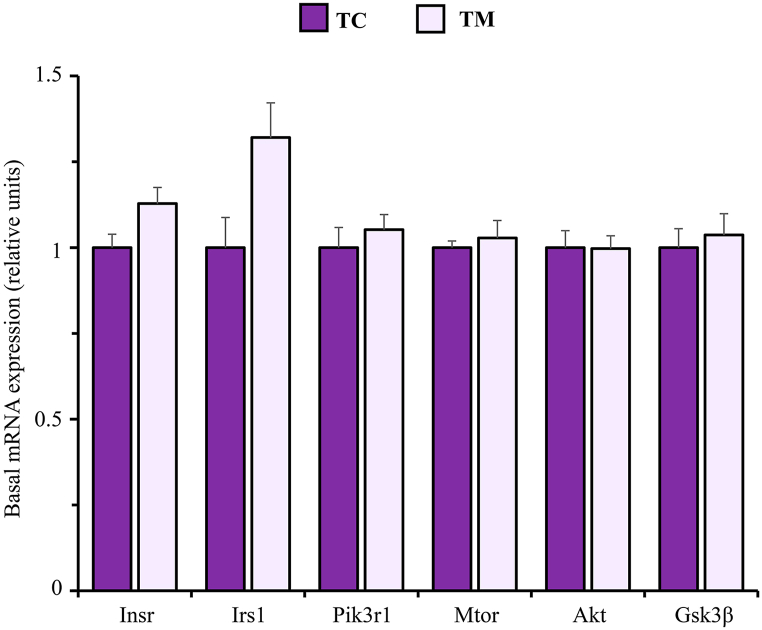
mRNA expression of key genes involved in the insulin signalling pathway in the frontal cortex of untreated (TC) and metformin treated (TM) Tau-VLW transgenic mice. 9-months-old Tau mice were treated daily with metformin for another 9 months and then mRNA expression of the indicated genes was measured as described in Experimental procedures. Data are represented as means ± SEM (TC, n = 5; TM, n = 6).

Fig. 6 shows that the gene expression of main regulators within the insulin signalling pathway measured (Insr, Irs1, Pik3r1, mTOR, Akt, and Gsk3β) was not significantly altered by metformin treatment. Nevertheless, the effect sizes showed a trend to mRNA levels of both Insr (d = 1.185) and Irs1 (d = 1.12) be slightly higher in metformin treated mice.

### 3.5 Effect of metformin on the relative expression of phosphorylated and total forms of key proteins involved in the insulin signalling pathway in the frontal cortex of Tau-VLW transgenic mice

Although metformin did not produce significant effects on the mRNA content of genes involved in the insulin signalling pathway, we also studied the effect of metformin treatment of Tau-VLW mice on the content of the total and phosphorylated forms of their respective gene products to assess their activation status.

**Fig. 7:**
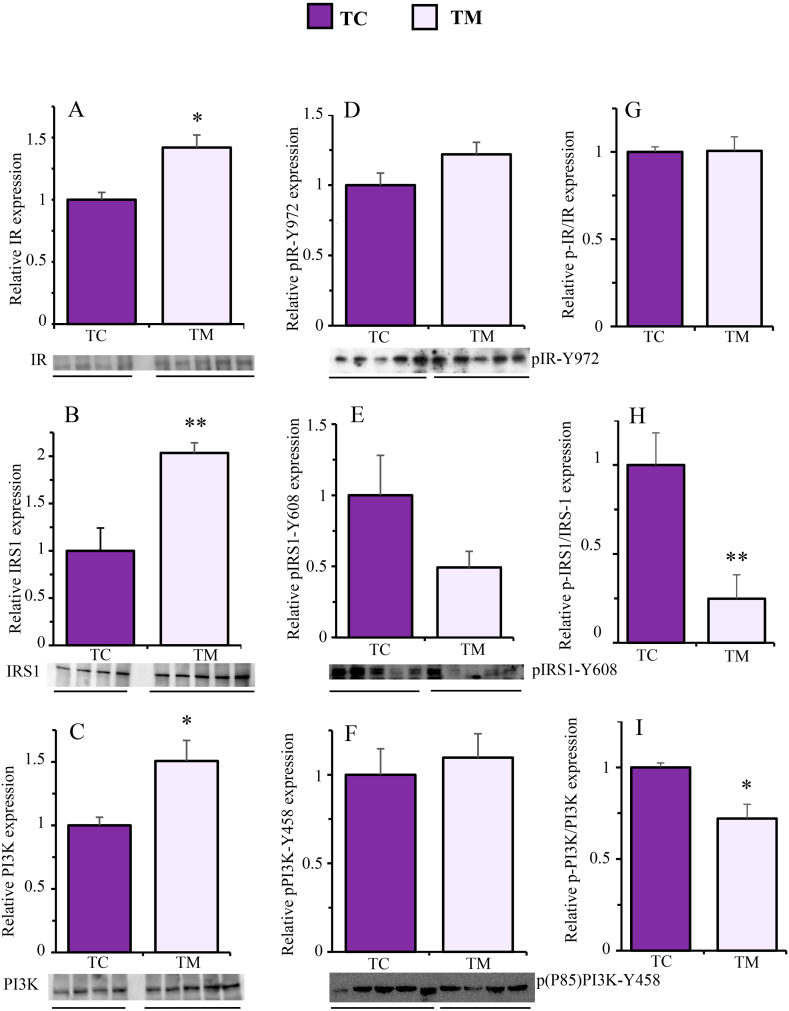
Effect of metformin on the expression of the total (A, B and C), phosphorylated (D, E and F) and active (G, H and I) forms of IR, IRS1 and PI3K, respectively, in the frontal cortex of Tau-VLW transgenic mice. 9-months-old Tau mice were treated daily with metformin for another 9 months and then the expression of the indicated proteins was measured by Western blot as described in Experimental procedures. Total and phosphorylated forms were normalized by β-actin (representative bands are showed in Supplementary Figs. 1 and 2). Phosphorylated forms normalized to the total forms are represented as active forms. Data are represented as means ± SEM (n = 4–5). Student's unpaired *t*-test was used to analyse significant differences. *p < 0.05, **p < 0.01. The full-length blot is provided in supplementary material as Supplementary Fig. 1 jpg and Supplementary Fig. 2 jpg.

Fig. 7 shows that metformin treatment significantly increased the total protein content of IR (Fig. 7A, p = 0.0125, d = 2.04), IRS-1 (Fig. 7B, p = 0.0039, d = 2.80) and PI3K (Fig. 7C, p = 0.0329, d = 1.78), but it did not produce significant effects on the expression of pIR-Y972 (Fig. 7D, p = 0.1168, d = 1.20), pIRS1-Y608 (Fig. 7E, p = 0.1100, d = 1.23), pPI3K–Y458 (Fig. 7F, p = 0.6426, d = 0.325). As observed in the same figure, when the ratios between the phosphorylated and total forms were calculated, the content of the active form of the IR was not affected by metformin (Fig. 7G, p = 0.9469, d = 0.052). However, metformin treatment significantly reduced the active forms of both IRS1 (Fig. 7H, p = 0.0068, d = 3.386) and PI3K (Fig. 7I, p = 0.0315, d = 2.262).

**Fig. 8:**
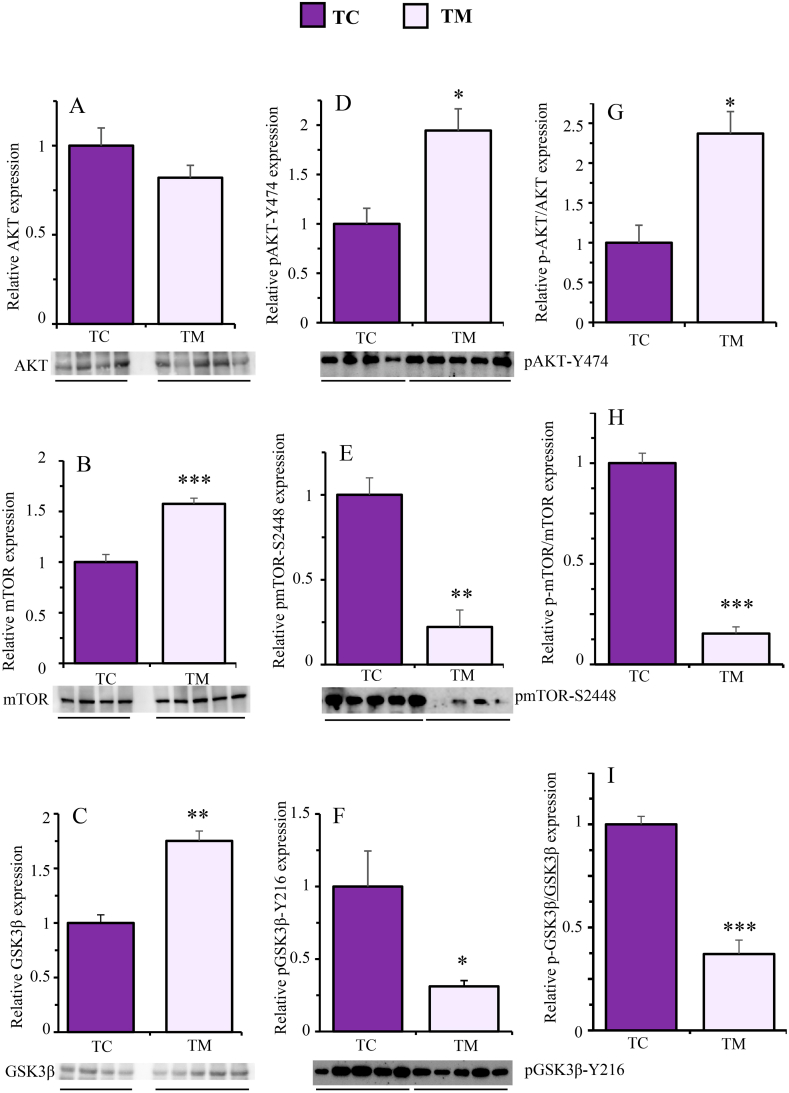
Effects of metformin on the expression of the total (A, B and C), phosphorylated (D, E and F) and active (G, H and I) forms of AKT, mTOR and GSK3β, respectively, in the frontal cortex of Tau-VLW transgenic mice. 9-months-old Tau mice were treated daily with metformin for another 9 months and then the expression of the indicated proteins was measured by Western blot as described in Experimental procedures. Total and phosphorylated forms were normalized by β-actin (representative bands are showed in Supplementary Figs. 1 and 2). Phosphorylated forms normalized to the total forms are represented as active forms. Data are represented as means ± SEM (n = 4–5). Student's unpaired *t*-test was used to analyse significant differences. *p < 0.05, **p < 0.01, ***p < 0.001. The full-length blot is provided in supplementary material as Supplementary Fig. 1 jpg and Supplementary Fig. 2 jpg.

Likewise, Fig. 8 shows that the metformin treatment did not affect the total protein content of AKT (Fig. 8A, p = 0.1698, d = 1.02) but it significantly increased that of mTOR (Fig. 8B, p = 0.0008, d = 3.63) and GSK3ꞵ (Fig. 8C, p = 0.0030, d = 6.93). By contrast, whereas metformin treatment significantly increased the phosphorylated form of AKT (Fig. 8D, *p* = 0.0130, d = 2.217), it did reduce the phosphorylated forms of mTOR (Fig. 8E, *p* = 0.0010, d = 13.4) and GSK3ꞵ (Fig. 8F, *p* = 0.0166, d = 2.1). The content of the active forms of these three proteins followed the same pattern as their phosphorylated forms. Therefore, the active form of AKT was significantly increased (Fig. 8G, *p* = 0.0114, d = 2.37) while those of mTOR (Fig. 8H, *p* = 0.0002, d = 11.42) and GSK3ꞵ (Fig. 8I, *p* = 0.0007, d = 5.59) were significantly reduced by metformin treatment.

## 4 Discussion

Our current study shows by [^18^F]FDG-PET neuroimaging that, at 12 months of age, Tau-VLW mice have brain glucose hypometabolism compared with WT mice. This result confirms previous findings showing that the transgenic tau-VLW mouse model of tauopathy [38,39] is characterized by an ageing-dependent brain glucose hypometabolism, starting at 11 months of age and becoming more evident in older animals and further correlating with histopathological markers of neurodegeneration in the brain areas overexpressing tau protein [37]. However, even though [^18^F]FDG-PET neuroimaging is a valuable tool for studying regional brain glucose metabolism reflecting regional activity, we cannot interpret glucose hypometabolism as direct evidence of central insulin resistance and further experiments would be necessary to directly investigate the occurrence of central insulin resistance in this model. Likewise, the changes in brain glucose metabolism can neither directly attributed to a particular cell type nor to be related to functional dedifferentiation or death, and despite the many attempts, the ultimate cause of brain glucose hypometabolism (such as neuronal death, reduced perfusion, etc) is yet to be known [3].

Our study further reveals that brain glucose hypometabolism in Tau-VLW mice is accompanied by signs of peripheral glucose metabolism dysfunction, as evidenced by fasting hyperglycemia, impaired responses to IpGTT, hyperinsulinemia, and a higher HOMA-IR index. The IpGTT is widely accepted as an appropriate method for administering glucose and the overnight fast is the most used in published studies (including ours) for glucose tolerance tests in rodents having the advantage of producing low, stable baseline blood glucose and insulin levels [47,48]. Likewise, the HOMA-IR index, whilst not being a replacement for direct measurement of insulin resistance, is considered as a widely accepted substitute [49].

Hyperglycaemia, hyperinsulinemia, and insulin resistance are hallmarks of T2DM and have been shown to increase the risk for various neurological diseases [50,51]. Consequently, the results from our first study gave allowed us to outline the tau-VLW mice as a potentially useful tool to study the complex reciprocal interrelationships between tauopathy and disorders related to central and peripheral glucose metabolism. Accordingly, we performed a second study focussed on the characterization of the long-term effects of metformin treatment in the tau-VLW transgenic mice. To this end we added metformin in the drinking water at a final concentration of 1 mg/mL. Our data do not support that metformin diluted in water produced a conditioned taste aversion because the water consumed by the Tau-VLW mice, both control (TC) and metformin (TM) was similar, being approximately 3 mL/day/mouse. The estimated daily water intake in an adult mouse can vary by more than 2-fold across strains [52] but 3 mL/day is within the normal range. Besides, other studies have shown that metformin diluted in water (3 g/L) does not alter water consumption in mice [53] and in our study metformin was diluted in water up to a of 1 g/L. Finally, metformin administration by oral gavage has been shown to induce conditioned taste aversion when administered at doses of 150 mg/kg or higher [54] but, in our study, the addition of metformin to the water in the TM group animals resulted in approximately 100 mg/kg/day.

Metformin is known to lower plasma glucose concentration by acting as an insulin sensitizer, improving fasting insulin levels and insulin-dependent hepatic glucose production, as well as increasing muscle glucose uptake, being one of the first-line antidiabetic drugs used for T2DM [55]. Given that metformin can cross the BBB [14], and taking into account its insulin-sensitizing properties [56], it seems reasonable to suggest that metformin may play a beneficial role in different types of dementia associated with insulin resistance, including AD, when considering the impaired brain glucose utilisation and energy production [57]. Metformin has been shown to reduce the risk of dementia [16] and, in combination with other drugs, improves working memory in diabetic patients [58]. Altogether metformin seems to be a particularly beneficial drug for the treatment of AD [59,60] as well as other neurological diseases [34,61]. Nowadays, the anti-hyperglycaemic and the insulin sensitizing effects of metformin added to its anti-apoptotic and anti-oxidative properties [[62], [63], [64], [65]], seems to have overall beneficial effects on aging and healthspan [66].

In this context, our results revealed that 9 months of metformin treatment (from 9 to 18 months of age) had a limited effect improving brain glucose metabolism based on the longitudinal [^18^F]FDG-PET neuroimaging studies. On one hand, this effect was restricted to the time-frame window age ranging from 11 to 15 months of age disappearing by the end of the experiment, when brain glucose hypometabolism was comparable between the two groups. On the other hand, unfortunately we do not have longitudinal data to correlate brain glucose metabolism with histopathological markers of neurodegeneration and phosphorylated tau protein levels. Despite these caveats, whether and, let alone to which extent, the temporal effect of metformin improving brain glucose metabolism from 11 to 15 months of age might contribute to the potential long-term effects of metformin needs to be further elucidated. Our current findings show that metformin improved glucose intolerance but did not alter insulin concentrations nor the HOMA-IR index, indirect markers of peripheral insulin resistance at 18 months of age in tau-VLW mice. No effects of chronic metformin treatment on insulin concentrations have been reported in female SHR mice [67]. Our results support a mild effect of metformin in improving peripheral glucose homeostasis, but it is interesting to notice that the treatment was well tolerated and that metformin-treated Tau-VLW mice were able to defend their BW throughout the experimental study. The results regarding mortality failed to reach significance, nevertheless, we consider important to notice that 5 of 12 TC mice (41.6 %) died before reaching the age of 18 months while only 2 of 13 TM mice (13.38 %) died before 18 months of age. In fact, it has been reported in SHR female mice, that the effects of metformin on survival and longevity are robust when the treatment started at 3 months of age, less evident when started at 9 months and absent when the treatment started at 15 months of age mice [67].

Overall, our results in line with the reported beneficial effects of metformin on ageing [24,66]. In fact, metformin seems to slow the ageing process by acting on mitochondrial metabolism and insulin signalling [55]. Studies done both in humans and in experimental animals have shown that dysregulation of insulin function promotes ageing and the development of neurodegenerative diseases [68]. Accordingly, insulin resistance and diabetes are considered to contribute to the development of the disease, mainly in the field of dementia [69].

Nonetheless we cannot disregard the fact that metformin is well known by its effect reducing BW by inducing the production of the growth differentiation factor 15 (GDF-15), a stress response cytokine that controls appetite and induces BW loss under various types of diseases [70]. Likewise, metformin has been shown to prevent BW gain and to improve glucose intolerance in mice fed a high-fat diet [53,71]. Future inquiries could shed some light on whether the effects of metformin are directed towards regulation of environment-integrated energy needs. In addition, GDF-15 has been involved in the full activation of AMPK [72]. Further studies are needed to determine whether the effects of metformin might involve an insulin-like effect by inducing a positive feedback loop between AMPK and GDF-15.

Unfortunately, in our study we neither measure AMPK nor tau phosphorylation. Activated AMPK is a tau kinase [73] that is found to be accumulated in pre-tangle- and tangle-bearing neurons in tauopathies [74]. In this context, metformin is known to activate AMPK but the reported effects of metformin on tau phosphorylation have been inconsistent. Thus, metformin attenuates diabetes-induced tau hyperphosphorylation by enhancing autophagic clearance [75], it reduces tau hyperphosphorylation, attenuating tau pathology and improving learning and memory deficits in the tau-seeded PS19 [76], and attenuates plaque-associated tau pathology in APP/PS1 mice [77]. However, it has been also reported that metformin promotes aggregation of neurofibrillary tangles mitigating the potential benefits arising from its tau dephosphorylating action in the tauopathy P301S mice model [29]. Thus, investigating the effects of metformin on phosphorylated AMPK as well as on phosphorylated tau will be of great interest in future studies to achieve a deeper and comprehensive understanding of the effects of metformin in this model.

Nevertheless, chronic metformin treatment significantly altered the profile of some of other key mediators of the insulin signalling transduction pathway mediated via the IR/IRS-1/PI3K/Akt/mTOR in the frontal cortex of 18-month-old tau-VLW mice, but only at post-transcriptional level. We also want to notice that the effects of metformin were evaluated at 18 months of age, after 9 months of metformin treatment, the results therefore providing a static scenario. Considering that treatment duration-dependent effects of metformin have been reported in a mice AD model [78], further longitudinal studies regarding the effects of metformin on key mediators of the insulin signalling transduction pathway will be of great interest.

In our study metformin increased the total IR content, but the absolute pIR-Y972 expression and its relative value to the total IR content where unchanged. Other studies have shown that intranasal metformin in mice reverses the effects of ICV-STZ-injection reducing pIR-Y972 in the hippocampus and the cerebral cortex of ICV-STZ-injected mice [79]. Likewise, metformin, administered (30 mg/kg/day) by oral gavage for 5 weeks increased pIR levels reduced by ICV-STZ-injection in adult albino rats ICV [80]. Our results show that metformin increased the total IRS-1 and PI3K content without affecting the expression of their phosphorylated forms pIRS1-Y608 and pPI3K–Y458. Nonetheless, the ratio between their phosphorylated and total forms was lower in the metformin-treated than in the untreated mice. The reduced active form of IRS-1 in Tau-TLW mice treated with metformin could be due to a shift in the phosphorylation of Y towards S residues induced by some downstream effectors of Akt, such as mTOR, S6K, JNK and GSK3, which are responsible for feedback inhibition of IRS-1 [[81], [82], [83]]. However, more studies are needed to decipher whether and to what extent metformin could alter the insulin signalling pathway by decreasing the phosphorylation of tyrosine residues and increasing the phosphorylation of serine residues in IRS-1.

Metformin treatment did not alter the content of Akt but it increased the expression of pAKT-Y474. It has been reported that full or partial activation of Akt, in addition to T308 phosphorylation (PDK1-dependent) and S473 phosphorylation (mTORC2-dependent), also requires phosphorylation at Y474, which is enhanced by receptor tyrosine kinases [84]. Interestingly, intranasal metformin in mice has been shown to reverse the effects of ICV-STZ-injection reducing pAkt-S473 in the hippocampus and cerebral cortices [79].

Chronic metformin treatment increased the total content of mTOR but it reduced the expression of pmTOR-S2448 as well as the ratio between its phosphorylated and total forms. Other study in diabetes-induced obesity mice has shown no effects of metformin [85]. Considering that signalling through the mTOR pathway has been related to rapid aging, progression of neurological diseases, as well as T2DM [86] our results would support a beneficial role of metformin. Likewise, metformin increased the content of GSK3 but reduced the expression of its phosphorylated active for pGSK3β-Y216 as well as the ratio between phosphorylated and total forms. This result is in agreement with previously reported effects of metformin in the Senescence-accelerated mouse-prone 8 (SAMP8) model of AD [87] and in STZ-injected Wistar rats [88]. It is well know that GSK3β is one the three main protein kinases strongly associated to the abnormal phosphorylation of Tau [89]. Interestingly, shutdown of GSK3β in transgenic tau-VLW mice with conditional overexpression of GSK3β in forebrain neurons, has been shown to lead to normal GSK3 resulting in normal phospho-tau levels, diminished neuronal death, and suppression of the cognitive deficit [90].

Herein we have shown that brain glucose hypometabolism measured by using [^18^F]FDG-PET neuroimaging, as well as the signs of peripheral glucose metabolism dysfunction are features of the transgenic tau-VLW mouse model of tauopathy making this model as a useful tool to study the mechanisms underlying the complex relationship between neurological disorders and glucose metabolism dysfunction. The effects of chronic metformin treatment modulating the insulin pathway in the cerebral cortex of tau-VLM mice are mainly found at post-transcriptional level. Especially important are the down-regulation of pIRS1-Y608 and pmTOR-S2448 and the up-regulation of pAKT-Y458, which in turn induces the phosphorylation and inhibition of pGSK3β-S9. Likewise, metformin inhibits the phosphorylation and activation of pGSK3β-Y216. Furthermore, our study shows that chronic treatment with metformin ameliorates brain glucose hypometabolism, improves peripheral glucose intolerance and appears to have a beneficial effect on the overall wellbeing of Tau-VLW mice. Altogether, our findings support that the use of metformin and other anti-diabetic drugs capable of crossing the BBB might be promising strategies for the prevention and/or treatment of neurodegenerative diseases characterized by dysregulation of insulin signalling in the brain.

## Funding

This work was supported by the Ramón Areces Research Foundation, PR2007_18/01, and the Spanish Ministerio de Ciencia e Innovación, Retos PID2019-106968RB-100. VH-C, YLBA and CNF have benefited from a research support contract financed by the Ramón Areces Foundation.

## Data availability statement

**Has data associated with your study been deposited into a publicly available repository?** ≫>No.

**Please select why**. ≫> Data will be made available on request.

## CRediT authorship contribution statement

**Verónica Hurtado-Carneiro:** Writing – review & editing, Visualization, Methodology, Investigation, Formal analysis, Data curation. **Yannick LeBaut-Ayuso:** Writing – review & editing, Visualization, Methodology, Investigation, Formal analysis, Data curation. **Esther Velázquez:** Methodology, Investigation. **Cinthya Flores-Lamas:** Visualization, Methodology, Investigation, Formal analysis, Data curation. **Rubén Fernández-de la Rosa:** Methodology, Investigation, Formal analysis, Data curation. **Luis García-García:** Writing – review & editing, Visualization, Validation, Supervision. **Francisca Gómez-Oliver:** Writing – review & editing, Visualization, Validation, Supervision. **Juan Miguel Ruiz-Albusac:** Writing – review & editing, Writing – original draft, Visualization, Validation, Supervision, Project administration, Investigation, Formal analysis, Data curation. **Miguel Ángel Pozo:** Writing – review & editing, Writing – original draft, Visualization, Validation, Supervision, Project administration, Funding acquisition, Conceptualization.

## Declaration of competing interest

The authors declare that they have no known competing financial interests or personal relationships that could have appeared to influence the work reported in this paper.

## Figures and Tables

**figs1:**
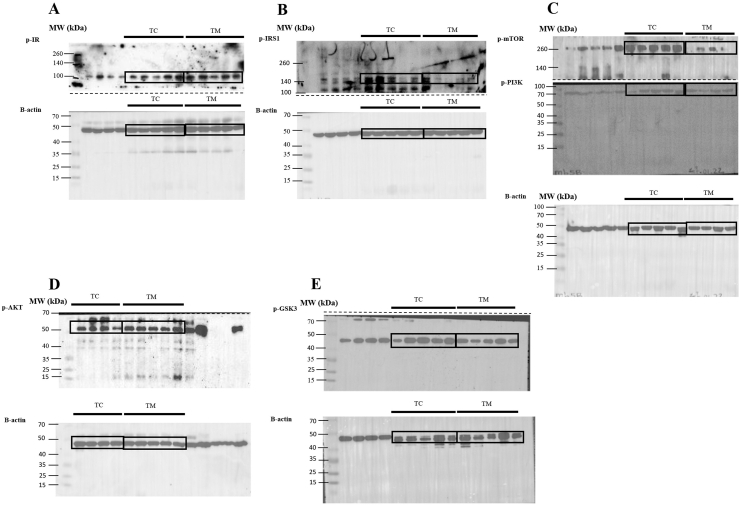


**figs2:**